# The Experiences of Australian School Mental Health Professionals during COVID-19 Lockdowns

**DOI:** 10.3390/children10071157

**Published:** 2023-07-01

**Authors:** Emily Berger, Grace Mackie, Andrea Reupert, Daliya Greenfeld, Kelly-Ann Allen, Fiona May, Gerald Wurf, Dianne Summers, Zoe Morris

**Affiliations:** 1 School of Educational Psychology and Counselling, Faculty of Education, Monash University, Clayton, VIC 3800, Australia; grace@leveluppsychology.com.au (G.M.); andrea.reupert@monash.edu (A.R.); daliya.greenfeld@monash.edu (D.G.); kelly-ann.allen@monash.edu (K.-A.A.); fiona.may@monash.edu (F.M.); gerald.wurf@monash.edu (G.W.); dianne.summers@monash.edu (D.S.); zoe.morris@monash.edu (Z.M.); 2 School of Rural Health, Faculty of Medicine, Nursing and Health Sciences, Monash University, Clayton, VIC 3800, Australia; 3 Centre for Wellbeing Science, Melbourne Graduate School of Education, University of Melbourne, Parkville, VIC 3010, Australia

**Keywords:** COVID-19, pandemic, students, mental health professional, psychologist, counsellor, therapy, assessment

## Abstract

Young people have emerged as one of the most impacted groups from the COVID-19 pandemic and related restrictions to daily activities, with disruptions to schooling, social interactions, and connections. Simultaneously, students’ access to school mental health professionals were restricted or modified. The aim of this paper was to identify how school mental health professionals supported and addressed the mental health needs of young people during COVID-19 restrictions in Australia. School mental health professionals were surveyed during the 2020 lockdowns using a questionnaire designed by researchers in the United States of America. The innovations school mental health staff adopted to support students during lockdowns and remote learning were presented, including telehealth services, digital resources, and the online training and support they received/provided. The barriers and facilitators to providing counselling and assessment services during lockdowns were identified, including issues with providing psychometric assessments during remote learning, and ethical concerns when delivering remote counselling to students. Recommendations have been included, which address how school mental health professionals could be supported to assess and treat young people during future pandemics and school restrictions.

## 1. Introduction 

### 1.1. The COVID-19 Pandemic

On 11 March 2020, the novel coronavirus 2019 (SARS-CoV-2; COVID-19) was declared as a global pandemic [1]. People infected with COVID-19 may require hospitalisation due to severe respiratory failure, while others present with milder symptoms, including shortness of breath, fever, and/or fatigue. To prevent the spread of COVID-19 and reduce the rates of infection and death, social distancing and lockdown laws were introduced across many districts. Australia introduced some of the most restrictive lockdown laws and measures in the world, including the closure of businesses, restaurants, sporting venues, schools, and universities, as well as the introduction of mandatory facemasks and social distancing [2]. Although these measures have reduced the morbidity and mortality rates associated with COVID-19, researchers have recorded increased psychological distress, family violence rates, substance abuse, and suicidality during the pandemic [3]. 

### 1.2. Psychological Impacts of COVID-19

A recent systematic review found that due to the COVID-19, severe acute respiratory syndrome (SARS), and Middle East respiratory syndrome (MERS) outbreaks, adults have reported suffering from increased depression, anxiety, and stress [4]. Another review by Brooks and colleagues (2020) found post-traumatic stress, confusion, and anger for adults and children quarantined during the SARS, MERS, Ebola, and H1N1 influenza outbreaks [5]. When parents were asked about the psychological impacts of COVID-19 and lockdown restrictions on their children, they reported increased hyperactivity, restlessness, inattention, anger, anxiety, and irritability [6,7,8]. Australian adolescents have reported suffering from a poorer mental health (e.g., anxiety and depression) since the beginning of the pandemic, with concurrent negative impacts on peer relationships, learning outcomes, and family relationships [9]. Moreover, Australian adults and adolescents with pre-existing mental health conditions have experienced significantly greater negative emotions (e.g., anxiety and depression) and negative wellbeing outcomes (e.g., sleep disturbance, psychological distress, and loneliness) during the pandemic, relative to the general population [9,10].

Parents and educators have reported similar effects of COVID-19 on their emotional wellbeing and their subsequent capacity to support children and students [11,12,13]. In a study of teachers’ descriptions of their work during the pandemic in Norway, Sweden, and the United States, COVID-19 school restrictions (e.g., online learning) were found to have caused significant worries about teachers’ health, potential job losses, and pay cuts [14]. Teachers from Canada and the United States reported elevated levels of psychological distress associated with high levels of concern for student wellbeing and an increased awareness of inequities among their students [15,16]. Similarly, a review of the impact of COVID-19 on healthcare workers found consistent reports of stress, anxiety, and depressive symptoms because of COVID-19 [17]. These psychological symptoms were found to be the most prominent amongst nurses, female workers, front-line healthcare workers, younger medical staff, and workers in areas with high COVID-19 infection rates.

### 1.3. COVID-19 and School Mental Health Care

School-based mental health professionals have always played a key role in supporting young people’s mental health and wellbeing within the school environment [18]. This has been achieved by providing both individual, student-level services, as well as broader system-level services [19]. Student-level services focus on evidence-based preventative and intervention strategies to manage student mental health concerns (e.g., counselling and therapies), skill building and development (e.g., social skills), assessment and testing (e.g., cognitive, and academic assessments), as well as vocational guidance (e.g., careers counselling) [19,20,21]. System-level services support young people through school-wide practices and policy development (e.g., developing anti-bullying policies), and by promoting the capacity of parents/caregivers and teachers to support young people [19]. In Australia, prior to the COVID-19 pandemic, most of these services were conducted in-person within the school environment.

Little is known about how school-based mental health professionals in Australia supported children and adolescents during the COVID-19 pandemic. To address a similar knowledge gap in the United States, Schaffer, and colleagues (2021) developed a 53-item survey that targeted full-time school psychologists employed in K-12 schools [22]. Based on the responses received, the authors found that psychologists faced difficulties, challenges, and changes to their roles and responsibilities, especially related to the types of services they offered during this period. While this study captured participants’ concerns and insights into how school psychology was impacted by COVID-19, given that COVID-19 restrictions varied between countries, it is likely that the impact of the pandemic on school-based mental health professionals is country-specific. Therefore, this study aimed to identify the experiences and practices of school mental health professionals (e.g., school psychologists and counsellors) during these COVID-19 restrictions in Australia, including support for at-risk or vulnerable young people. There are indications that access to online and telephone counselling increased during the pandemic [23,24,25]; however, it is unclear what (if any) adaptations were made by school-based mental health practitioners in response to the pandemic, school closures, and online learning restrictions. Given there have been seven international health crises in the last 20 years [26], the outcomes of this research can be used to guide mental health service delivery in schools during other pandemic or disaster events also causing school closures and lockdowns. The research questions for this study included:

1. What online practices did Australian school-based mental health professionals employ to support young people’s mental health during these COVID-19 restrictions, including identifying and supporting the students who were at risk?

2. What were the barriers and facilitators in addressing students’ mental health needs during these COVID-19 restrictions in 2020?

3. What do school-based mental health professionals recommend in regard to assessment and treatment innovations for students during future pandemics or similar events?

## 2. Materials and Methods

### 2.1. Sampling and Recruitment

A purposive sampling method was used to recruit participants who worked as mental health professionals within Australian schools, including psychologists, counsellors, social workers, and wellbeing team members. After ethics approval from the Monash University Human Research Ethics Committee was provided, digital flyers and notices advertising the research project and a link to complete the survey were distributed amongst the personal networks of the research team via professional associations (e.g., the Australian Psychological Society), and through paid social media advertisements (including Facebook, LinkedIn, Instagram, and Twitter). Paid advertisements were targeted toward individuals based on their school-based mental health occupation Australia-wide.

### 2.2. Participants

A total of 105 participants responded to this survey. After data cleaning was completed, 20 respondents were subsequently removed due to either not providing consent to participate or providing no further data outside of consenting to participate. Therefore, the final sample consisted of 85 participants aged between 24 and 59 years old, respectively (*M* = 38.11 years, *SD* = 8.12 years). Of this sample, 92.69% of participants identified themselves as women and 7.32% identified as men. The majority of participants were registered as psychologists with the Psychology Board of Australia (75.61%), with counsellors (15.85%), social workers (3.66%), provisionally registered psychologists with the Psychology Board of Australia (3.66%), and wellbeing staff (2.44%) also being represented within this sample. Participants’ years of experience in their various school-based roles ranged from 1 to 26 years (*M* = 6.44 years, *SD* = 6.46 years).

The majority of the sample reported working in either Victoria (55.41%) or New South Wales (33.78%), with Queensland (5.41%), Tasmania, (2.70%), Western Australia (1.35%), and the Australian Capital Territory (1.35%) also being represented in this sample. Most participants reported working in metropolitan schools (75.31%), while the rest worked in regional schools (19.75%) and rural/remote schools (4.94%). Over one quarter of participants (28.05%) reported being in Stage 4 lockdown at the time of completing the survey, with a further 15.85% in Stage 3, 23.17% in Stage 2, and 7.32% in Stage 1 (see below for a description of these stages). Furthermore, nearly one-quarter (23.17%) of participants were unsure of what COVID-19 lockdown restriction stage they were in, while a further 1.22% reported being under no restrictions. Most participants’ primary location of work at the time of completing the survey was either at school (41.46%) or at home (40.24%), with a further 18.29% working in a combination of both home and school settings.

Restrictions and stages of lockdown varied between the different Australian states. Stage 1 restrictions included the ban of non-essential outdoor gatherings of 500 or more people, indoor gatherings of more than 100 people, and restricting visitors to aged care. Stage 2 included closure of non-essential services, and people were encouraged to work from home. Stage 3 included people only being allowed to leave home for four reasons, including shopping for food and essential supplies, receiving medical care, exercising, and attending work. Moreover, teaching and learning were moved online for prep (first year of school) to Year 10 students for those under Stage 3. Years 11 and 12 students continued to attend classes at school, and vulnerable students (e.g., students with a disability) were able to be supervised at school. Stage 4 included further restrictions, such as people not being allowed to travel more than 5 km from home without a permit, a curfew from 8 pm to 5 am, and exercise in outdoor public spaces being limited to only one hour per day. At Stage 4, schools were closed to learning, with all teaching and learning activities for students from prep to Year 12 conducted online. However, vulnerable students were still able to be supervised in school.

### 2.3. Materials

#### Online Survey

The online survey used in the current study was initially developed by a United States-based research team [22] and was modified to suit the Australian context with permission from the authors. All participants were asked to base their responses on the delivery of school psychologist services at the peak of the pandemic, and to consider the impacts to their services during remote learning. Participant demographics were collected, which included age, gender identity, state or territory location of work, professional role within schools, years of experience in their current role, COVID-19 restrictions in place at their location, and primary location of work (i.e., school, home, or school and home) during the COVID-19 restrictions.

The detailed research questions and a review of peer-reviewed articles in the areas of crisis intervention, distance learning, tele-counselling, and previous pandemic impacts were all integrated and used to develop the survey items [22]. The survey sought to determine: (1) the general types of psychological support and services delivered to students during the COVID-19 restrictions (three items: one ‘select all that apply’ item and two open-ended items); (2) the delivery of counselling services to students during the COVID-19 restrictions (three open-ended items); and (3) the delivery of psychological assessments to students during the COVID-19 restrictions (three open-ended items). For more information about the survey, please refer to Reupert and colleagues (2021) and Schaffer et al. (2021) [22,27].

### 2.4. Procedure

After clicking on the link provided via the digital flyers, participants were presented with the explanatory statement to the study. A consent button was presented at the end of this document. Clicking on this button indicated informed consent to participate in the study. Participants were then presented with the online survey. The survey was open for participant completion from 18 May 2020 to 26 October 2020. Victorian schools were closed for most of May to October, New South Wales schools were closed from around April to June, and the other states experienced shorter school closure periods from around March to April 2020, respectively.

### 2.5. Data Analysis

Survey data were analysed using IBM Statistical Package for Social Sciences 26 (SPSS-26). Descriptive statistics were calculated for all the demographic variables of interest to our study, including frequencies and percentages for the categorical and dichotomous variables, and means and standard deviations for the continuous variables. The open-ended items were analysed using Braun and Clarke’s (2006) six-phase approach to thematic analysis [28]. These phases include: (1) familiarisation with the data; (2) initial coding; (3) searching for themes; (4) reviewing themes; (5) defining and naming themes; and (6) finalising analysis write-up. Two authors independently analysed the open-ended data using thematic analysis. The themes developed by both authors were then compared, and a final set of themes was developed by the authors. The open-ended data were then coded to determine the number and percentage of participants who provided a response related to each theme. After coding, descriptive statistics were then used to determine the number and percentage of participants who responded to each theme.

## 3. Results

The results are presented under each of the three research questions for this study.

**RQ 1: What online practices did Australian school-based mental health professionals employ to support young people’s mental health during these COVID-19 restrictions, including identifying the students who were at risk?**

Participants were asked to select the ways in which they had been delivering social-emotional, behavioural, or academic support services to students during the COVID-19 restrictions from the options listed in Table 1. Responses were received from 77 of the 85 participants and are summarised in Table 1.

As presented in Table 1, the most common social-emotional, behavioural, and academic online support services delivered to children during these COVID-19 restrictions were telehealth interventions or tele-counselling, with 86% of participants providing this service. The next most provided support, reported by 29% of participants, was the use of online databases to post/share social-emotional, behavioural, or academic support content with students. This was closely followed by sharing videos with parents/caregivers about the common social-emotional, behavioural, or academic issues faced by children (26%). Finally, 21% of participants utilised other support services delivered via emails to students and parents/caregivers through the provision of articles and resources posted on school websites or online wellbeing hubs, along with the upskilling of staff to respond to students’ needs through the delivery of online professional development and participation in individual education plan (IEP) meetings.

Table 2 presents participants’ responses to the open-ended question ‘Have you and/or your school undertaken any novel strategies to support student wellbeing given COVID-19 restrictions? If yes, please specify’. Overall, 18 participants responded no, 13 selected unsure, 42 said yes, and 12 participants did not respond, respectively. Participants who responded with yes provided an open-ended response of the novel strategy they used to support student wellbeing.

As shown in Table 2, there were several novel strategies adopted by school mental health practitioners to support student wellbeing during the COVID-19 restrictions. The most common strategy adopted by participants was to provide telephone, email, and online support services for students. This included both group and individual student online support, resources, activities, and telephone calls regarding students’ welfare and wellbeing, as well as emails sent to students. This was followed by the next most common strategy being adopted by 17% of participants, which was online staff training and support. Less frequently noted, but still noted as novel strategies, were changes to the student curriculum and workload (12%), the utilisation of student wellbeing surveys (10%), and support for and engagement with students’ parents/caregivers (7%).

Participants were then asked, ‘Have you and/or your school undertaken any novel strategies to support the identification of at-risk students? If yes, please specify’. Thirty-three participants responded no, 26 responded yes, 12 responded unsure, and 14 participants did not respond, respectively. The thematic analysis results from the 26 participants who responded yes to this question and provided an open-ended response are presented in Table 3.

Table 3 presents the novel strategies that were employed by school mental health practitioners to identify the at-risk students. The most common strategies included administering surveys and self-assessments to students (31%), contacting parents of students (31%), and developing and implementing whole school approaches (31%), such as consistent wellbeing policies and processes. These were followed by the next most utilised strategies, including monitoring student attendance and engagement with school (23%), managing the referrals of students (19%), and other strategies (15%), such as having teachers/mentors visit student homes to drop off schoolwork, liaising with year level coordinators, and encouraging peer-to-peer contact and support (4%).

**RQ2: What were the barriers and facilitators in addressing students’ mental health needs during these COVID-19 restrictions?**

Table 4 presents the barriers that have challenged school mental health practitioners in conducting psychological assessments with students during the COVID-19 restrictions. Participants were asked ‘Given COVID-19 restrictions, what are three barriers (if any) that have challenged you as a school psychologist/counsellor to assess students?’ A total of 31 participants responded to this question. The most common barrier faced by 39% of participants was the inability to assess students face-to-face to accurately evaluate their psychological needs. The next most common barriers faced by 12% of participants were issues related to students’ assessment in terms of adapting the content of the assessment to an online platform, lack of referrals for the assessments, and advice to cease assessments during the COVID-19 restrictions. Less frequently noted, but still identified as barriers to assessing students, were technical difficulties in performing assessments online (19%), lack of practitioner knowledge or ability to assess students online (16%), time constraints (13%), lack of parent collaboration (10%), issues with confidentiality/privacy (10%), and individual student factors (13%), such as lack of attendance and poor behaviour. Other barriers (26%) included staff lack of supervision, lack of clear directives for assessments, and all assessments ceasing during the lockdowns.

Participants also identified several facilitators to assessing students during the COVID-19 restrictions. Participants were asked ‘Given COVID-19 restrictions, what are three things (if any) that have helped you as a school psychologist/counsellor to assess students?’ Nineteen participants responded to this question. As presented in Table 5, the most common facilitators reported by 21% of participants were having access to peer support and supervision, as well as access to alternative forms of communication to contact students and parents (e.g., online platforms, telephones, and through the internet; 37%). The next most common facilitators reported by 16% of participants were collaboration with parents and having enough time to complete their assessments (16%). Less commonly reported facilitators were having access to technology, such as iPads for assessment purposes (11%), the implementation of workplace policies and procedures (11%), adequate or suitable workspaces to conduct assessments (11%), and other reasons (11%), such as collaboration with teams and supervisors. It should be noted that 16% of participants reported that they did not conduct assessments during the COVID-19 restrictions, and thus were unable to provide insight into facilitators. Additionally, a further 11% of participants did not identify any facilitators to the assessment of students.

Facilitators to provide counselling services are presented in Table 6. Participants were asked ‘Given COVID-19 restrictions, what are three things (if any) that have helped you as a school psychologist/counsellor to counsel students?’ Of the 85 participants, 40 provided a response to this question. The most common facilitator was access to online platforms, which was reported by 50% of participants. The next most common facilitator was having access to a supportive team within the school (38%), including other mental health practitioners, teachers, and employers. Following this, 23% of participants reported that having access to digital resources functioned as a facilitator in providing counselling services to students. Less commonly reported facilitators included access to supervision and peer consultation (18%) and other facilitators (13%), such as having an appropriate office space at home and having prior familiarity with online platforms.

Participants were asked ‘Given COVID-19 restrictions, what are three things (if any) that have challenged you as a school psychologist/counsellor to counsel students?’ Of the 85 participants sampled in this study, 56 provided a response to this question. As presented in Table 7, the most common barriers to providing counselling to students during the COVID-19 restrictions were issues with student attention and engagement on online platforms, reported by 29% of participants. The next most common barrier, reported by 25% of participants, was issues with confidentiality/privacy of students due to being within the family home. This was followed by technology and internet connection issues, which was reported by 23% of participants. Less commonly reported barriers included lack of face-to-face interactions (20%) and other barriers (14%), such as client age and capacity to engage in online counselling.

**RQ3: What do school-based mental health professionals recommend in regard to assessment and treatment innovations for students during future pandemics or similar events**?

Participants were asked ‘Given COVID-19 restrictions, what are three things (if any) your school could do to support you to assess students?’ Overall, 32 participants provided a response to this question. As presented in Table 8, the most common recommendations of support schools could provide to enable school mental health practitioners to assess students was the access to suitable and relevant resources (25%), such as appropriate technology and forms of communication. The next most common recommendations of support were being provided with access to additional support (19%), such as peer consultation and supervision, and being provided with funded resources (16%) to assist with assessments. Less frequently reported recommendations of support were being provided with funded professional development (6%), schools being open to change and flexibility regarding the administration of assessments (6%) and developing policies and procedures to support students in novel ways (3%). Thirteen percent of participants reported that they already felt supported by their schools to assess students, while some participants (13%) reported not being sure of how the school could support assessments with students.

Participants were then asked ‘Given COVID-19 restrictions, what are three things (if any) your employer could do to support you to counsel students?’ Thirty-nine participants responded to this question. As presented in Table 9, the most common recommendation of support employers was increased wellbeing and support offered to school mental health practitioners (21%). This included regular staff wellbeing check-ins and recognising staff burnout. The next most common recommendations of employer support were increased role flexibility (15%) and establishing better referral processes (15%). Thirteen percent of participants already felt supported by their employers, while 10% reported that nothing could be recommended as the barriers were outside of their employers’ control (e.g., childcare access for their own children). A smaller percentage of participants recommended access to IT support (10%), access to supervision (10%), and access to training in telehealth (5%). Thirteen percent of participants reported other recommendations, such as a more consistent communication between staff and assistance with interpreters for parents.

## 4. Discussion

This study explored the experiences and practices of Australian school mental health professionals during the COVID-19-related lockdowns and school closures. Use of telehealth/tele-counselling interventions to replace face-to-face counselling was a significant change made by participants. Participants also reported providing more online support and professional development to their colleagues during these lockdowns. Others have highlighted an increase in online counselling services (e.g., tele-counselling, online group intervention/workshops, and telephone sessions), as well as online support/resources (e.g., communication media) in international educational settings during these COVID-19 lockdowns and/or restrictions [29,30]. Schaffer et al. (2021), as well as Reupert et al. (2021) reported comparable results, which confirmed that an increasing number of psychologists shifted to remote delivery methods to ensure the continuation of psychological services [22,27].

While there are many advantages and strengths to online counselling and telehealth services (e.g., ability to socially distance, and reduced travel time) [31], researchers have also reported on various issues for professional and ethical practice associated with online counselling services. Common issues include risks to client/patient privacy and confidentiality (e.g., cyber-security), matching the mode of care to the client/patient (e.g., some health issues are less suitable for online services), health professional competency to deliver services using an online platform/system, and the ability to respond to the risk or emergency issues (e.g., disclosure of suicidal thoughts or intentions) [32,33]. The results from the current study supported these findings within the context of schools and working with young people, highlighting that issues of privacy, confidentiality, and online competency were also experienced within the telehealth services offered in schools.

The adoption of remote practices for students deemed to be at risk of developing severe mental health conditions was another innovation reported by the participants. This included the use of online surveys and self-assessment tools, greater contact with parents of at-risk students, and adoption of new policies to manage the high needs of these students. Reflecting on the ethical and professional practice standards of remote mental health service delivery for vulnerable students, it is crucial for school mental health professionals to be aware of the potential practice and ethical challenges that may arise from this work [34]. For instance, privacy and confidentiality may be breached when attending counselling from within the home/family environment, which may work against at-risk students engaging with the online counselling services. Long standing research has shown that adolescents in general are reluctant to access mental health services due to barriers, such as stigma and confidentiality concerns [35,36]. The impact of tele-counselling on students’ help-seeking intentions and behaviour was outside of the scope of this study. However, reports that mental health providers were concerned about confidentiality with students may have exacerbated students’ reluctance to seek help during the pandemic.

Other ethical and professional issues were also noted in the results of the current study. Participants reported that a common barrier to supporting students during the pandemic was their inability to provide face-to-face services. One of the services provided by school mental health staff that was most severely impacted by the pandemic was their ability to conduct psychological assessments with students. Participants reported that several assessments ceased during the pandemic. Research and standards are lacking for practitioners who are seeking to improve their skills to conduct online assessments with clients. Krach and colleagues (2020) found that assessment publishers acted quickly to offer computer-adapted, tele-assessment methods; however, these methods were developed using minimal empirical data, and test publishers and government agencies often recommended using tele-assessments methods with caution [37]. Additionally, other studies have highlighted the issues associated with inadequate training for school mental health professionals to use and access tele-assessments [38,39], suggesting that further professional training and development is required to ensure that school mental health professionals are able to competently use online assessment methods.

Our results suggest there is still significant work to be completed to improve the confidence, knowledge, and skills of the practitioners delivering tele-assessment tools. Professional development and protocols are needed to assist mental health professionals to feel confident and equipped to administer, interpret, and make recommendations for students’ mental health based on online assessments. It is possible that tertiary students, who completed their mental health training and practicums during the pandemic, will be more confident and prepared to deliver and interpret online psychological assessments compared to experienced practitioners, who have limited experience with these methods of assessments. However, more research is needed to explore the aspects of validation, interpretation, and reporting of psychological assessment tools delivered online [38].

While participants noted issues concerning the conduct of online assessments during the pandemic, and a lack of confidence delivering online assessments, participants acknowledged that colleagues provided a great deal of support around the remote delivery of assessments and counselling during the pandemic. Online platforms were also deemed to be more useful for participants in terms of providing counselling services to students during the pandemic and school closures compared to administering assessments. This study found that it was much easier for practitioners to shift to an online delivery mode of counselling compared to the online administration of assessments during the pandemic. However, consistent with the prior literature, the challenges associated with maintaining client confidentiality/privacy, matching online services to the client’s needs, making sure professionals are competent in their use of online services, and developing protocols for responding to risk or emergency situations [34,38,40] were all raised by practitioners in Australia.

To address these issues, the Australian Psychology Society (APS) developed recommendations for providers of telehealth measures under the Better Access initiative. These recommendations provide information and resources to assist psychologists in safely and adequately utilising telehealth measures, such as implementing online security measures, assessing client suitability for online services, completing risk assessment and management, training in the use of telehealth, and adjustments to the informed consent and referral policies [41]. While these considerations are relevant in addressing some of the issues that participants faced when delivering online counselling services, they fail to address the ethical and practical issues that are associated with online assessment administration. This suggests that Australian school psychologists still lack the appropriate guidance and resources to assist the administration of online assessment services. It is reasonable to assume that restrictions and school lockdowns will occur in the future in response to COVID-19, other pandemic events, or other disaster events [26]. Thus, continued research and practice protocols concerning the appropriate and evidence-based administration of assessment tools during lockdown periods are required. Research on the impacts for students, schools, and families on the lack of access to psychological assessments over the course of Australia’s 2020 and 2021 lockdowns is also needed.

These recommended considerations from the APS (2022) are largely consistent with the recommendations offered by participants in the current study in terms of the delivery of remote assessment/counselling for students [38]. For example, in line with the APS document, many participants recommended better telehealth training, adjusting referral policies and procedures for students, better IT support, and more general resources for telehealth services. Participants also recommended an additional focus on staff wellbeing and support, as well as additional supervision, which were not specifically outlined by the APS. Thus, in order for school psychologists to continue providing remote services to students, it will be important for schools to ensure that school mental health practitioners are well supported both personally and professionally during future lockdown or remote working circumstances.

## 5. Limitations

This study is the first known study to explore the experiences of school mental health staff in response to students during the COVID-19 pandemic in Australia. However, the small sample size limits the generalisability of the results. Unfortunately, there is a lack of known data on the gender and occupation distribution of school mental health professionals in Australia. However, data on the gender distribution of psychologists in Australia, working across a variety of a settings, shows that our data were skewed towards female rather than male participants. Data were also skewed towards the Victorian and New South Wales-based mental health providers; however, national data shows that Victoria and New South Wales have the highest number of registered psychologists compared to the other states [42]. The open-ended questions used in the survey were able to capture the perceptions and experiences of school mental health practitioners; however, these questions increased the length of the overall survey. It is likely that the additional time and effort required of participants to respond to these questions resulted in some participants choosing to not provide a response. Furthermore, analysing qualitative responses through a questionnaire did not permit probing and more detailed responses as would be the case for an interview or focus group data. For example, a few participants stated they had not adopted novel strategies to support students, including at-risk students, during the pandemic, and it was unclear from their responses why this was the case. The qualitative data captured also did not permit statistical comparisons between practitioners from the different states and regions in Australia, between mental health practitioners who have distinct roles in schools, or staff from different education sectors (e.g., primary compared to secondary school). The results of this study indicated that older students were more able to engage with practitioners using telehealth and other remote services compared to the younger students. Therefore, the experiences of professionals in secondary schools may be different to those working in primary schools. The different lockdown and school closure restrictions imposed by these different states also potentially impacted the comments and expertise of participants. Mental health practitioners from Victoria, which experienced the longest lockdowns in Australia, were deemed to be more likely to have developed greater expertise and adopt more remote online practices during the 2020 lockdowns, compared to professionals from other states.

## 6. Conclusions

The current study was the first known piece of empirical research to explore how school mental health professionals addressed and supported the mental health needs of young people during the COVID-19 restrictions in Australia. These findings demonstrated that school mental health professionals relied upon and adopted online and telehealth services to support young people during the COVID-19 restrictions. Whilst access to online platforms/services, additional staff support, and professional supervision were amongst the facilitators to supporting young people’s mental health needs, issues, such as technology/internet difficulties, confidentiality and privacy concerns, and online student engagement were all found to act as barriers to supporting young people. The concerns raised by school mental health providers should be considered when developing future resources and professional learning activities to ensure that suitable and evidence-based psychological support can be provided to school students.

## Figures and Tables

**Table 1:** Social-emotional, behavioural, or academic online support services delivered to students amid COVID-19 restrictions.

Response	N (*n* = 77)	%
Telehealth interventions or tele-counselling via webcam or phone	66	86
Use of database such as Google classroom to post social, emotional, behavioural, or academic support	22	29
Developing or posting videos on common social, emotional, behavioural, or academic issues that parents/caregivers can use with/on their children (psychoeducation)	20	26
Mailing packets or newsletters with social, emotional, behavioural, or academic interventions	18	23
Other	16	21

Note: The percentage adds up to more than 100%, as participants may have selected more than one option.

**Table 2:** Novel strategies introduced to support student wellbeing.

Theme	N (*n* = 42)	%
Phone, email, and online support for students	42	100
Online staff training and support	7	17
Student wellbeing survey	4	10
Changes to student curriculum	5	12
Support for and engagement with parents/caregivers	3	7

Note: The percentage adds up to more than 100%, as participants may have reported more than one option.

**Table 3:** Strategies to identify at-risk students.

Theme	N (*n* = 26)	%
Survey and self-assessments for students	8	31
Contact with parents	8	31
Peer support	1	4
Whole school approaches	8	31
Monitoring attendance and engagement	6	23
Referral management	5	19
Other	4	15

Note: The percentage adds up to more than 100%, as participants may have reported more than one option.

**Table 4:** Barriers to assessing students.

Theme	N (*n* = 31)	%
Time constraints	4	13
Confidentiality/privacy	3	10
Inability to assess face-to-face	12	39
Technical difficulties to perform assessment online	6	19
Lack of ability and knowledge to assess online	5	16
Issues with the content of the assessment and their adaptation to online mode	8	12
Interface with school	8	26
Parent collaboration	3	10
Individual student factors	4	13
Other	8	26

Note: The percentage adds up to more than 100%, as participants may have reported more than one option.

**Table 5:** Facilitators to assessing students.

Theme	N (*n* = 19)	%
Access to alternative communication	7	37
Access to technology to perform assessments	2	11
Workplace policies and procedures	2	11
Adequate/suitable workspace	2	11
Collaboration with parents	3	16
Having enough time	3	16
Peer support/supervision	4	21
Did not conduct assessments during COVID-19	3	16
None	2	11
Other	2	11

Note: The percentage adds up to more than 100%, as participants may have reported more than one option.

**Table 6:** Facilitators to providing counselling to students.

Theme	N (*n* = 40)	%
Access to online platforms	20	50
Access to digital resources	9	23
Supportive team	15	38
Supervision and peer consultation	7	18
Professional development	10	25
Other	5	13

Note: The percentage adds up to more than 100%, as participants may have reported more than one option.

**Table 7:** Barriers to providing counselling to students.

Theme	N (*n* = 56)	%
Technology and internet issues	13	23
Issues with student attention and engagement	16	29
Lack of face-to-face interactions	11	20
Issues with confidentiality/privacy	14	25
Other	8	14

Note: The percentage adds up to more than 100%, as participants may have reported more than one option.

**Table 8:** Recommended support to assess students.

Theme	N (*n* = 32)	%
Access to resources	8	25
Funded professional development	2	6
Funded resources	5	16
Additional support	6	19
Openness to change/flexibility	2	6
Policies and procedures to support students in new ways	1	3
Already supported	4	13
Other	4	13

Note: The percentage adds up to more than 100%, as participants may have reported more than one option.

**Table 9:** Recommended support to counsel students.

Theme	N (*n* = 39)	%
IT support	4	10
Training in telehealth	2	5
Increased flexibility	6	15
Increased staff wellbeing and support	8	21
Provide supervision	4	10
Better referral process	6	15
Already felt supported	5	13
Nothing	4	10
Other	5	13

Note: The percentage adds up to more than 100%, as participants may have reported more than one option.

## Data Availability

Not available.
